# The effect of staircase stopping accuracy and testing environment on stop-signal reaction time

**DOI:** 10.3758/s13428-022-02058-1

**Published:** 2023-01-26

**Authors:** Dominic M. D. Tran, Nahian S. Chowdhury, Justin A. Harris, Evan J. Livesey

**Affiliations:** 1 School of Psychology, The University of Sydney, Camperdown, NSW Australia; 2 Neuroscience Research Australia, Randwick, NSW Australia

**Keywords:** Inhibition, Stop-signal task, Staircase adjustment, Strategic slowing, Testing environment

## Abstract

**Abstract**

The stop-signal task is widely used in experimental psychology and cognitive neuroscience research, as well as neuropsychological and clinical practice for assessing response inhibition. The task requires participants to make speeded responses on a majority of trials, but to inhibit responses when a stop signal appears after the imperative cue. The stop-signal delay after the onset of the imperative cue determines how difficult it is to cancel an initiated action. The delay is typically staircased to maintain a 50% stopping accuracy for an estimation of stopping speed to be calculated. However, the validity of this estimation is compromised when participants engage in strategic slowing, motivated by a desire to avoid stopping failures. We hypothesized that maintaining stopping accuracy at 66.67% reduces this bias, and that slowing may also be impacted by the level of experimenter supervision. We found that compared with 50%, using a 66.67% stopping accuracy staircase produced slower stop-signal reaction time estimations (≈7 ms), but resulted in fewer strategic slowing exclusions. Additionally, both staircase procedures had similar within-experiment test–retest reliability. We also found that while individual and group testing in a laboratory setting produced similar estimations of stopping speed, participants tested online produced slower estimates. Our findings indicate that maintaining stopping accuracy at 66.67% is a reliable method for estimating stopping speed and can have benefits over the standard 50% staircase procedure. Further, our results show that care should be taken when comparing between experiments using different staircases or conducted in different testing environments.

**Supplementary Information:**

The online version contains supplementary material available at 10.3758/s13428-022-02058-1.

Response inhibition is critical for healthy functioning. However, measuring response inhibition is not straightforward. Reaction time (RT; e.g., from a key press) provides a behavioral index that can operationalize the fluency of cognitive processing, but successfully inhibiting a response provides no simple measurable outcome (because no key was pressed). Substantial efforts have been made by many researchers to measure response inhibition, with a particular focus on quantifying individual differences in inhibitory control using behavioral tasks that can be easily administered. The ability to inhibit or cancel an action is typically measured using the stop-signal task (SST; Lappin and Eriksen, 1966; Logan and Cowan, 1984; Vince, 1948), which cleverly uses *unsuccessful* inhibition to estimate response inhibition capacity. The SST is a reliable (Weafer et al., 2013) and well-validated (Nichols & Waschbusch, 2004) task that is widely used. For example, poor performance on the SST has been associated with attention-deficit hyperactivity disorder (Oosterlaan, Logan, & Sergeant, 1998) and obsessive–compulsive disorder (Menzies et al., 2007), as well as increased risk for obesity (Nederkoorn et al., 2007) and gambling disorder (Chowdhury et al., 2017).

In the SST, participants are required to make an observable response to a cue on “go” trials. The means of engaging the response can vary, such as a key press to an imperative cue (Verbruggen et al., 2008) or a timed key release (Coxon et al., 2006). On a minority of “stop” trials, participants are prompted to inhibit this overt motor response via the presentation of a stop signal in advance of, or in parallel with, the go cue. Throughout the task, the delays between the initiation of the go motor response and stop signal can be either predetermined (e.g., method of constant stimuli) or adjusted via staircasing. Most contemporary versions of the SST use some method of staircase adjustment to determine the delay between the response cue and the stop signal. That is, whether or not a participant successfully cancels their response on the current stop trial determines *when* the stop signal is presented on the next stop trial. In a stepwise staircasing procedure, if the participant successfully stops, the next stop trial is made more difficult by presenting the stop signal later in the trial (increasing what is known as the stop-signal delay, or SSD, between the response cue and the stop signal), and thereby providing less time to stop if the process of responding has already been initiated. In contrast, if the participant fails to stop, the next stop trial is made less difficult by presenting the stop signal earlier in the trial (decreasing the SSD between the go cue and the stop signal), and thereby providing more time to stop before the process of responding is completed.

The staircasing procedure of adjusting the SSD on a (stop) trial-by-trial basis provides an effective method for estimating an individual’s stop-signal reaction time (SSRT); a measure of how quickly one can cancel their response. When matched for response speed, participants who can successfully stop following longer SSDs will have fast SSRT, while participants who require shorter SSDs to successfully stop will have slow SSRT. There are a number of ways to estimate SSRT from the staircase parameters (Verbruggen & Logan, 2009) but this paper is focused on the staircasing procedure itself. The exact method of SSD adjustment can vary across different versions of the SST. For example, some researchers have implemented a Bayesian staircase procedure that typically requires fewer trials to accurately estimate an individual’s SSRT (Livesey & Livesey, 2016; Weise et al., 2018). However, the most common method of SSD adjustment is to simply increase or decrease the delay by a set numerical value (e.g., ± 50 ms) after each stop trial depending on the success of that trial. This method of symmetrical SSD adjustment maintains stopping accuracy at approximately 50% for each individual, regardless of their stopping speed.

Adjusting SSD to achieve a 50% stopping accuracy has been recommended as the standard protocol for reliably estimating SSRT in a consensus review (Verbruggen et al., 2019). However, there are situations in which maintaining a 50% stopping accuracy is less optimal. A common scenario is when participants are motivated to perform well on stop trials. In reaction to failed stop trials, participants will often *strategically slow* their response speed in anticipation of late stop-signal trials. When participants deliberately slow their responding to avoid commission errors (i.e., unsuccessful stopping), this leads to increased Go RT, and as a consequence, an inaccurate estimate of SSRT (Verbruggen et al., 2013). To avoid this, some experimenters remind their participant throughout the experiment that they should not slow down their go responses, and it is recommended that blocks of trials with evidence of substantial slowing be excluded from analyses (Verbruggen et al., 2013). Excluding blocks of data is a common by-product of the 50% stopping accuracy staircase that not only costs time and resources but may also reduce the reliability of that individual’s SSRT estimate. One possible solution to counteract strategic slowing is to adopt a staircase that maintains a higher percentage stopping accuracy. A higher stopping accuracy will allow participants to achieve more successful stops, and it is plausible that this experience will result in less slowing.

Testing environment is another potential factor that can influence a participant’s propensity for strategic slowing. Completing the SST comes with competing self-motivations to perform well by reducing stop failures and task-demands to respond as quickly as possible as instructed by the experimenter. However, the SST is widely used in scientific research and clinical assessments where the balance of these demands can vary. For example, in one-on-one testing environments, there is strong motivation from task-demands to respond as quickly as possible when the experimenter can individually remind participants after each block not to slow down. In comparison, in online testing environments, there may be less direct pressure to respond quickly, and participants can more freely self-determine how fast or slow they want to respond. Additionally, we note that online testing is becoming increasingly popular, particularly since the COVID-19 pandemic. Therefore, while a staircase stopping accuracy greater than 50% may reduce the amount of strategic slowing, the extent to which participants engage in strategic slowing may also interact with the testing environment.

To investigate the impact of staircase stopping accuracy on competing self-motivations and task-demands for engaging in strategic slowing, the present study assessed the validity and within experiment test–retest reliability of SSRT estimation using a procedure for maintaining stopping accuracy at 66.67% compared to the commonly used 50%. We hypothesize that using a 50% staircase procedure will result in more data exclusions due to increased strategic slowing compared with using a 66.67% procedure. To assess the impact of testing environment on the measurement of stopping, the present study compared the validity and within experiment test–retest reliability of SSRT estimation collected from individual laboratory testing, group laboratory testing, and online testing. Based on the level of experimenter supervision to motivate task engagement and adherence to instructions, we hypothesize that participants tested in the laboratory will have more comparable SSRT estimates compared with participants tested online, with participants tested online having slower SSRT estimates.

## Method

### Participants

Participants were undergraduate students from the University of Sydney who participated for course credit as part of their first- or second-year psychology course. Informed consent was obtained before commencing the experiment and all procedures were approved by the University of Sydney Human Research Ethics Committee.

Experiment 1A included data from 113 participants (mean age = 20.37, SD = 3.08). We aimed to collect at least 40 participants in each group but did not reach our target minimum sample size in one group due to face-to-face testing restrictions because of the COVID-19 pandemic. Data were collected from 42 participants for individual laboratory testing, 31 participants for group laboratory testing, and 40 participants for online testing. The sample size was determined using G-Power for a repeated measures, between factors ANOVA with two measurements (50%, 66.67%) and three groups (individual laboratory, group laboratory, and online testing). To detect a medium effect size f = 0.25 at an error rate α = .05 with 80% power, a total sample of 120 participants was needed (40 participants per group). Two participants from the online testing group were excluded due to poor task adherence: one had a mean stop accuracy of 0% and one had a mean stop accuracy of 22.25% (this participant did not attempt the experiment seriously, only adhering during some trials of the 66.67% condition and none from the 50% condition).

Experiment 1B included data from 219 participants (mean age = 19.90, SD = 2.50). We aimed for approximately 80 per group in the group testing and online conditions, and tested as many as possible in the individual testing condition over the same time period, noting that data collection is much more time-consuming and resource intensive in this condition. This resulted in us collecting data from 61 participants for individual laboratory testing, 76 participants for group laboratory testing, and 84 participants for online testing. We only have 59 data files from the individual laboratory testing group as the server failed to save two data files due to a power outage. Due to the way online sessions were offered in batches, we ended up with four extra data sets for the online testing group. Four participants from the online testing group were excluded due to poor task adherence: one had a mean stop accuracy of < 1% and three made no responses.

### Design

Staircase adjustment was a within-participant factor with two levels. All participants completed the SST with two staircases, one maintaining stopping accuracy at 50% and one at 66.67%. The testing environment was a between-participant factor with three levels. Individual laboratory testing involved having one participant per experimenter present. Group laboratory testing involved having between two and four participants per experimenter present. Online testing involved no direct experimenter supervision. Experiments 1A and 1B were identical except for the programs used to administer the SST (detailed in the next section).

### Apparatus

All groups completed a unimanual visual SST, based on the STOP-IT program (Verbruggen, Logan & Stevens, 2008). In Experiment 1A, participants in the two laboratory testing groups (individual and group) completed the experiments on PC with the task programmed in MATLAB. Participants in the online testing group completed the experiment on their own computer or laptop running any suitable operating system and program compatible with JavaScript. The online SST was programmed in jsPsych (modified from https://github.com/fredvbrug/STOP-IT) using the same parameters and stimuli as the MATLAB version. Participants accessed the task via a link using a web browser. In Experiment 1B, participants from all three testing environment groups (individual, group, and online) completed the jsPsych SST to control for any differences in stimulus presentation timing between programs.

### Procedure

The task stimuli were presented against a mid-grey background. The two go signals were a black arrow pointing left or a black arrow pointing right. Participants were asked to press the left arrow key with their right index finger when they saw the left arrow, or press the right arrow key with their right middle finger when they saw a right arrow. Arrows were presented at the center of the monitor and had a height of 1.7° and a width of 1.5° visual angle viewed from approximately 75 cm from the screen. On some trials, a stop signal (a blue square – 0.7° x 0.7° of visual angle) would appear after the arrow cue, indicating that participants were required to stop their initiated response. They were informed that on some of the stop trials, the blue square would appear early and it would be easier to stop, but on other trials the stop signal would appear late and it would be difficult to stop. Participants were also instructed to respond as quickly and accurately as possible to the arrow, and not to delay their responses in anticipation of the stop signal. Task instructions were displayed on screen and the experimenter provided no further verbal instructions in the individual or group testing conditions, so instructions were matched for all groups.

On each trial, a black fixation dot (0.2° x 0.2° of visual angle) was presented for 500 ms, followed by a go signal for 1500 ms, which represented the maximal response time. The presentation of the stop signal depended on the staircasing procedure condition. There were four phases of the stop-signal task – two phases used the 50% stopping accuracy staircasing procedure, and two phases used the 66.67% stopping accuracy staircasing procedure. Phases alternated between the 50% and 66.67% staircase, the starting staircase was randomized across participants, and the staircase was reset at the beginning of each phase. There was also a short practice phase of 32 trials with trial-by-trial feedback before the experimental phase, which did not present feedback.

On stop trials, the initial SSD was set at 250 ms. For the 50% staircase phases, following stop trials where inhibition was successful, the SSD was increased by 50 ms, and after trials where inhibition was unsuccessful, the SSD was decreased by 50 ms. This staircase ensured an overall stopping accuracy rate of approximately 50%. For the 66.67% staircase phases, following stop trials where inhibition was successful, the SSD was increased by 25 ms; and after trials where inhibition was unsuccessful, the SSD was decreased by 50 ms. This staircase ensured an overall stopping accuracy rate of approximately 66.67%. That is, the stopping accuracy staircase is approximated by Eq. (1).

1 $$Staircase\ accuracy=\frac{\mid \Delta \textrm{SSD}\ \textrm{post}\ \textrm{unsuccessful}\ \textrm{stop}\mid }{\mid \Delta \textrm{SSD}\ \textrm{post}\ \textrm{unsuccessful}\ \textrm{stop}\mid +\mid \Delta \textrm{SSD}\ \textrm{post}\ \textrm{successful}\ \textrm{stop}\mid }$$

Notably, since monitor refresh rates are most commonly 60 Hz, this was set as the monitor refresh rate for all testing conditions (matching the online group). Because the 60-Hz refresh rate has a refresh cycle time of 16.67 ms, a 25-ms change in the SSD could result in a 33.33-ms change in the stimulus onset asynchrony (SOA) between the go and stop signals. This will only affect some SSDs (e.g., 125 or 175 ms), and won’t impact SSDs that are multiples of 50 ms (e.g., 150 or 200 ms). Therefore, the actual SOA between go and stop signals could be 8.33 ms longer than the intended SSD on 50% of trials for the 66.67% staircase, resulting in the SSD being presented 4.17 ms later, on average. This means that the average ΔSSD post successful stop is 29.17 ms and therefore the 66.67% staircase condition will tend towards a slightly lower stopping accuracy of 63.16%. Both staircase procedures converged close to their respective stopping accuracy (see Table 1). Relatedly, the mean SSD recorded for the 66.67% staircase will be approximately 4.17 ms shorter than the actual SSD presented to participants and SSRTs for the 66.67% staircase will be overestimated by this same amount. However, given that SSRTs are rarely compared across experiments and that the absolute SSRT value is not as meaningful as the variance across individuals or experimental conditions, the validity of a 66.67% staircase can be more appropriately assessed by the SSRT correlation comparing 50 vs. 66.67% rather than the SSRT mean.

Each phase consisted of 192 trials split into two blocks of 96 trials, and each block contained 25% stop trials. Between the two blocks, there was a 15-s break where performance feedback for the most recent block was displayed, including: average Go RT, average stopping accuracy, number of incorrect go responses, and number of missed go responses. Between each phase there was a self-timed break and performance feedback for the most recent block was displayed.

The integration method (Verbruggen & Logan, 2009; Verbruggen et al., 2013) was used to calculate SSRT for each block (of 96 trials) separately for each participant. Each phase contained two blocks and the mean SSRT for each phase was the average SSRT across two blocks (or one block when the other block was excluded). This technique involves subtracting the mean SSD from the *n*th Go RT, whereby *n* represents a point on the Go RT distribution in which the integral of the RT curve is equivalent to p(respond|signal). The *n*th Go RT is the point on the RT distribution that separates the “fast RTs”, which represent the probability of failed stopping, from the “slow RTs”, which represent the probability of successful stopping. Another popular method of calculating SSRT is to subtract the mean SSD from the mean Go RT. However, given the higher accuracy for the 66.67% staircase, the mean SSD is expected to be shorter than for the 50% staircase. In this case, subtracting the SSD from mean Go RT would lead to slower SSRT estimates in the former, and therefore the SSRT estimates would not be comparable between the conditions. Thus, using the integration method corrects for this issue, as the *n*th Go RT should be faster in the 66.67% staircase than in the 50% staircase (see Verbruggen et al., 2013 for more information on the mean versus integration method). Go omissions were not included in the estimate of SSRT. Given the arrow stimuli were displayed for 1500 ms and that overall accuracy was very high (see Results), including go omissions resulted in a very small difference (across the four phases, before excluding for strategic slowing, there was a mean increase of 1.89 ms in SSRT). However, for experiments with shorter response deadlines or more complicated response cues that result in a larger proportion of go omissions, it is recommended to use a replacement method (Verbruggen et al., 2019).

Data were excluded by block if the mean stop accuracy was < 25%. Using a 25% stop accuracy cut off is recommended by Verbruggen et al. (2019) and was approximately 3 standard deviations below the mean stopping accuracy of the 50% staircase procedure. Data were also excluded if the horserace assumption was violated within a phase (mean Go RT < mean Stop RT) or if mean SSRT was < 100 ms for the phase (Verbruggen et al., 2019). Data were also excluded if the mean Go accuracy was < 70%. As previously mentioned, SST data are often excluded when there is evidence of increasing Go RT during a block. Verbruggen et al. (2013) demonstrated that an increase of 1.5 ms per go trial led to a significant difference in the SSRT estimate relative to the “actual” SSRT estimate. We adopted a similar but more conservative value of 2 ms per go trial, such that any block with a slowing slope greater than this threshold was excluded (Verbruggen & Logan, 2009; Verbruggen et al., 2013). Although Go RTs can be used as a proxy for strategic slowing, they are biased against slow responders. Hence, changes in Go RT across trials as measured by slowing slopes is a more sensitive measure of strategic slowing that is specific to increases in RT due to the task.

### Analysis

Data were analyzed as a (2) x 3 factor ANOVA with staircase (50%, 66.67%) as a within-participant factor, and testing environment (individual, group, online) as a between-participant factor. Planned orthogonal contrasts compared the laboratory testing environment (individual + group) versus online testing environment [1, 1, –2], as well as individual lab versus group lab testing against each other [1, –1, 0]. The data were also complemented with Bayesian analysis using JASP (Version 0.12.2) with default priors and interaction effects reported across matched models (i.e., “BFincl”, indicating the BF for the model including the interaction term against matched models without the interaction term).

## Results

Due to the differences in software used between groups in Experiment 1A, results from Experiment 1A and 1B are aggregated only for the within participant factor of staircase (50%, 66.67%); any possible effect of software will affect both staircases. Results for the between participants factor of testing environment (individual, group, online) are presented only for Experiment 1B.

### 50% versus 66.67% staircase accuracy

Given that staircase was a within-participant factor, we present the aggregated descriptive statistics for stopping accuracy and SSRT across Experiments 1A and 1B in Table 1. Mean Go accuracy averaged across both 50% staircase phases was 98.18% (SD = 2.77, range = 79.86–100%) and both 66.67% staircase phases was 98.18% (SD = 2.71, range = 81.94–100%).

**Table 1:** Descriptive statistics of stopping accuracy and SSRT (separated by inclusion and exclusion of strategic slowing) aggregated from Experiments 1A and 1B for 50% and 66.67% staircases

	Stopping accuracy		SSRT inc. slowing		SSRT exc. slowing	
	50%	66.67%	50%	66.67%	50%	66.67%
*n*	320	320	320	320	288	298
Missing	12	12	14	14	46	36
Mean	0.518	0.646	242.369	248.601	243.887	251.050
Std. Dev.	0.053	0.068	41.168	38.599	45.109	38.701
Minimum	0.333	0.385	152.292	146.750	137.750	152.979
Maximum	0.677	0.896	439.865	398.667	456.938	390.354

### Staircase × testing environment

**Fig. 1:**
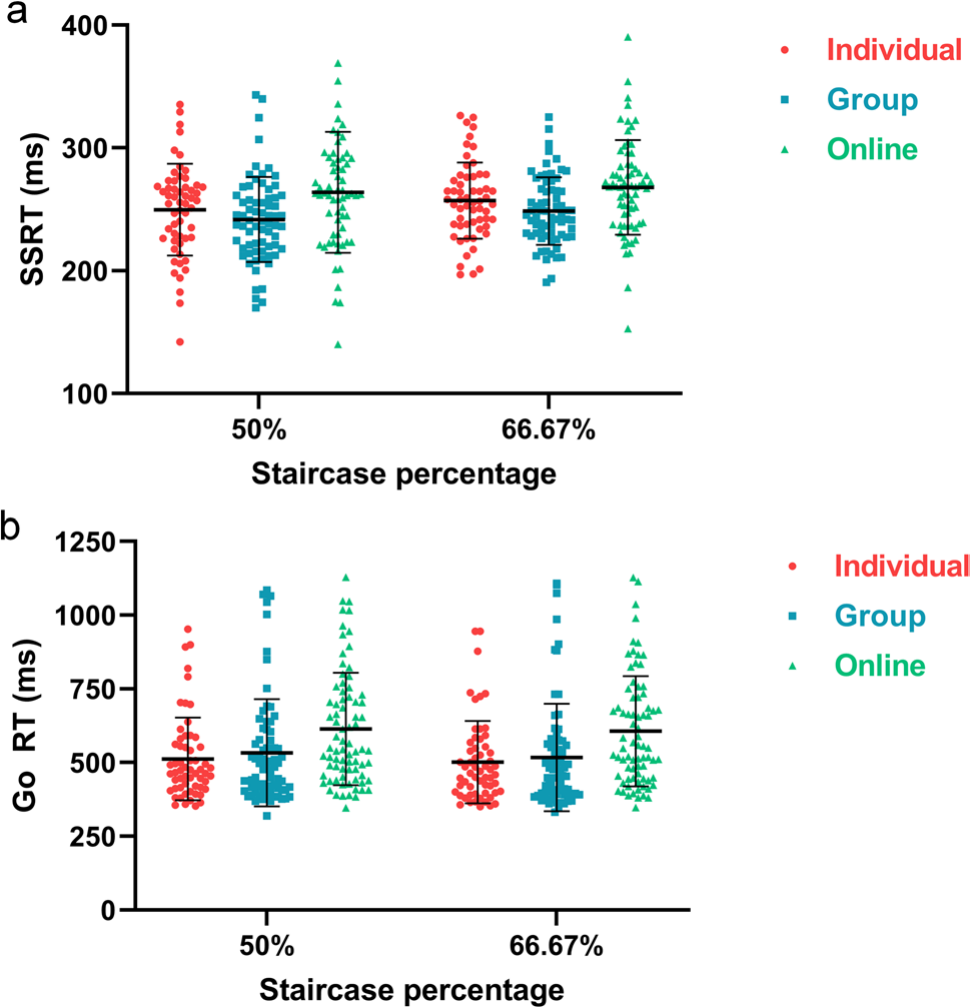
Individual data plots of a) stop-signal reaction time and b) go reaction time for Experiment 1B. *Lines* represent mean and standard deviation. *Dots* represent individual data points

The effect of testing environment was analyzed exclusively for Experiment 1B where all participants completed the SST using the same program. Individual data plots of SSRT and Go RT for each condition are shown in Fig. 1. Experiment 1A (see Supplementary Materials) produced similar results to Experiment 1B but there was a larger effect of testing environment on SSRT. Given that the testing environment in Experiment 1A was confounded with SST program (MATLAB for individual and group; jsPsych for online), the testing environment results (main, interaction, simple effects) from Experiment 1A are difficult to interpret.

#### Stop-signal reaction time

Descriptive statistics of SSRT after excluding for strategic slowing (> 2 ms/trial slowing slope) are presented in Table 2. Using a 66.67% stopping accuracy staircase resulted in significantly slower SSRT estimates (F(1,180) = 10.75, *p* < .001, η_p_^2^ = 0.06, BF_10_ = 16.88, error % = 0.73). There was an overall effect of testing environment on SSRT estimates (F(2,180) = 6.33, *p* = .002, η_p_^2^ = 0.07, BF_10_ = 15.87, error % = 1.61), and no staircase × environment interaction (F(2,180) = 0.85, *p* = .852, η_p_^2^ = 0.002, BF_incl_ = 0.07). Follow-up orthogonal contrasts revealed that there was a significant effect between laboratory testing (individual + group) compared to online testing (t(180) = 3.28, *p* = .001), and no difference between individual compared to group laboratory testing (t(120) = 1.22, *p* = .225).

**Table 2:** Descriptive statistics of stop-signal reaction times (ms) for Experiment 1B

	50% stopping accuracy			66.67% stopping accuracy		
	Individual	Group	Online	Individual	Group	Online
*n*	57	69	64	58	69	69
Missing	2	7	20	1	7	15
Mean	249.689	241.687	263.850	257.189	248.566	267.967
Std. Dev.	37.423	34.645	49.195	31.043	27.536	38.501
Minimum	142.083	169.958	140.104	196.823	190.500	152.979
Maximum	335.313	343.083	456.938	326.375	325.063	390.354

Stop-signal reaction time statistics are presented and analyzed by first averaging across blocks (96 trials) within a phase before taking the average of the two phases (192 trials) from each staircase adjustment (50 vs. 66.67% stopping accuracy). In the case where one block is excluded, the phase is also excluded because there are insufficient stop trials to estimate SSRT (Verbruggen et al., 2019). In the case where one phase is excluded, the value from the other phase is given a 100% weighting. In the case both phases are excluded, the value is labeled as missing

#### Go reaction time

Descriptive statistics of Go RT are presented in Table 3. Using a 50% staircase adjustment resulted in significantly slower Go RT compared to using a 66.67% staircase adjustment (F(1,203) = 8.53, *p* = .004, η_p_^2^ = 0.04, BF_10_ = 6.23, error % = 0.99). Testing environment also had a significant effect on Go RT (F(2,203) = 7.30, *p* < .001, η_p_^2^ = 0.07, BF_10_ = 21.80, error % = 2.69), and there was no staircase × environment interaction (F(2,203) = 0.52, *p* = .595, η_p_^2^ = 0.005, BF_incl_ = 0.08). Follow-up orthogonal contrasts revealed that there was a significant difference between laboratory testing (individual + group) compared to online testing (t(203) = 3.80, *p* < .001), and no significant difference between laboratory testing (t(129) = 0.67, *p* = .505).

**Table 3:** Descriptive statistics of go reaction times (ms) for Experiment 1B

	50% stopping accuracy			66.67% stopping accuracy		
	Individual	Group	Online	Individual	Group	Online
n	59	73	77	59	73	76
Missing	0	3	7	0	3	8
Mean	512.486	533.048	614.023	501.662	517.221	606.753
Std. Dev.	140.372	181.784	190.930	139.743	182.052	186.994
Minimum	352.270	319.197	346.436	350.536	331.268	347.854
Maximum	952.886	1084.443	1128.694	945.092	1108.033	1127.633

Go reaction time statistics are presented and analyzed taking the average of the two phases (192 trials) from each staircase adjustment (50 vs. 66.67% stopping accuracy). In the case where one phase is excluded, the value from the other phase is given a 100% weighting. In the case both phases are excluded, the value is labeled as missing

#### Strategic slowing

**Fig. 2:**
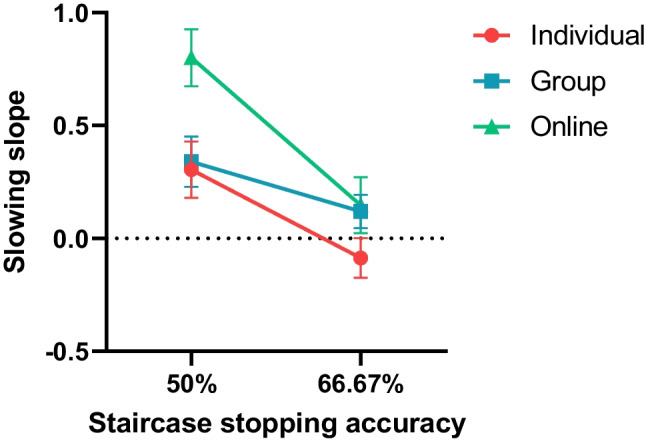
Mean slowing slopes (ms/trial) for staircase accuracy by testing environment. *Error bars* represent SEM

The Go RT findings reveal that using a 50% staircase resulted in significantly slower *overall* task responding compared to using a 66.67% staircase. Additionally, we assessed the impact of staircase on slowing *within* blocks by analyzing the change in Go RT across trials as a linear slope. The mean slopes for each condition are shown in Fig. 2. Using a 50% staircase adjustment resulted in more slowing (steeper slope) compared to using a 66.67% staircase adjustment (F(1,203) = 28.63, *p* < .001, η_p_^2^ = 0.12, BF_10_ = 155,506.78, error % = 0.87). Testing environment also had a significantly effect on response slowing within phases (F(2,203) = 4.15, *p* = .017, η_p_^2^ = 0.04, BF_10_ = 1.07, error % = 0.88), and there was a significant staircase × environment interaction (F(2,203) = 3.46, *p* = .033, η_p_^2^ = 0.03, BF_incl_ = 1.43). Follow-up orthogonal contrasts revealed that there was a significant difference between laboratory testing (individual + group) compared to online testing (t(203) = 2.75, *p* = .007), and no significant difference between laboratory testing (t(129) = 1.03, *p* = .306).

As described in the Method section, the presence of strategic slowing was defined as an increase of 2 ms per go trial or more, and blocks meeting this threshold were excluded from the analysis. Counting the number of blocks excluded based on this criterion, using a 50% staircase adjustment resulted in nearly double the number of blocks (43 vs. 19) classified as affected by strategic slowing (and therefore more blocks being excluded) compared to using a 66.67% staircase procedure. This distribution is significantly different to what would be expected if the rate of exclusions were equally distributed amongst the two types of staircase procedures (χ^2^ (1, *N* = 62) = 9.29, *p* = .002).

### Reliability

Reliability analysis was aggregated for Experiments 1A and 1B. The within experiment test–retest reliability of SSRT estimates across phases was assessed with a correlation analysis. The two phases using a 50% staircase procedure were significantly correlated (*n* = 293, *r* = .55, *p* < .001), as were the two phases using a 66.67% staircase procedure (*n* = 286, *r* = .54, *p* < .001). Average SSRT estimate from the 50% staircase procedure was significantly correlated with average SSRT estimate from the 66.67% staircase procedure (*n* = 317, *r* = .75, *p* < .001). Additionally, Table 4 presents the within experiment test–retest reliability of the two staircase procedures by testing environment. As a supplementary analysis (Table S2), we also computed the reliability of each staircase procedure before strategic slowing exclusion.

**Table 4:** Descriptive statistics of within experiment test–retest reliability by testing environment were analyzed exclusively for Experiment 1B

	50% stopping accuracy			66.67% stopping accuracy		
	Individual	Group	Online	Individual	Group	Online
*n*	40	43	37	43	51	43
*r*	.60	.55	.60	.68	.41	.64
*p*	< .001	< .001	< .001	< .001	.003	< .001

## Discussion

The current study assessed the validity and reliably of using a 66.67% staircase procedure compared with a 50% stopping accuracy across three different testing environments. We found that using a 66.67% staircase procedure resulted in slower SSRT estimates (≈ 7 ms) compared to using a 50% staircase procedure. We found that using the recommended 50% staircase procedure resulted in more overall slowing of Go RT, more strategic slowing within blocks, and consequently more blocks meeting criterion for exclusion based on excessive slowing. Finally, maintaining stopping accuracy at 50% and 66.67% led to comparable levels of within experiment test–retest reliability. In terms of testing environment, we found that overall Go RTs were slower for online testing compared with laboratory testing. Similarly, estimates of SSRT were slower for online testing compared with laboratory testing.

Our results show that using a 66.67% staircase procedure generates similar SSRT estimates to those produced using a 50% staircase procedure albeit slightly slower. This difference in SSRT is partly due to a 4.17 ms overestimation of SSRT when using a 66.67% staircase on a 60-Hz monitor.^1^ Absolute differences in SSRT may be a problem when directly comparing between experiments that use different staircases, although between experiment comparisons are seldom good practice as they include various extraneous variables. Importantly, the small effect of increasing SSRT estimates does not restrict using a 66.67% staircase for analyses within experiments as it is the variance in SSRT across individuals and experimental conditions that matter most for measuring stopping speed, such as for correlations with other individual difference measures, or comparisons of different conditions. Most critically, stopping speed estimates from the 50% and 66.67% staircase procedures were highly correlated with each other (*r* = .75). Given the within experiment test–retest reliability of each staircase^2^, this proportion of shared variance between the two is about as high as can be expected, suggesting that the two staircases measure the same underlying construct. We found a large within experiment test–retest correlation of greater than *r* = .50, and this was true for both the 50% and 66.67% staircase procedures. Our findings show that the validity and reliability of SSRT estimates using a staircase procedure that maintains stopping accuracy at 66.67% is comparable to that using the recommended 50% staircase procedure.

On measures of slowing, using a 66.67% staircase procedure can have advantages over using the standard 50% staircase. A common result of using a 50% staircase is that participants will strategically slow their response in anticipation of a stop trial to be more likely to successfully inhibit their response. As predicted, we found that participants were significantly slower on go trials when stopping accuracy was maintained at 50% accuracy. Moreover, strategic slowing slopes were significantly steeper within the 50% staircase blocks, and this had a meaningful (undesirable) consequence of leading to more blocks being excluded from the 50% staircase data. After exclusion, the within experiment test–retest reliability (comparing Tables 4 and S2) of the 50% staircasing procedure increased by *r* = .18, averaged across testing environment; the 66.67% staircasing procedure also increased by *r* = .18, averaged across testing environment. Therefore, not excluding blocks with strategic slowing weakens to reliability of the SSRT estimate, but using a 66.67% staircase requires less data to be excluded to reach a comparable level of reliability compared with using a 50% staircase.

Adjusting a 66.67% staircase compared with a 50% staircase was particularly helpful in reducing strategic slowing for participants in the online testing environments. For participants in the laboratory testing environment, the reduction in slowing slopes when using a 66.67% staircase compared with a 50% staircase was numerically smaller compared with the online testing environment (Fig. 2). The findings reveal that using a 66.67% staircase resulted in less strategic slowing than the 50% staircase, but also that rates of strategic slowing was more comparable across testing environments.

Completing the SST often generates conflicting self-motivations to perform well by avoiding errors and to adhere to the instructed task demand to respond quickly. The former often motivates participants to strategically slow responding, while the latter requires participants to speed up responding. Our results indicate that varying staircase stopping accuracy and the testing environment in which participants complete the SST had a main and interactive effect on managing these competing motivations. We found that the conventional 50% staircase poorly manages these conflicting goals and was problematic in leading to more data exclusions. In comparison, the 66.67% staircase better managed competing motivations by reducing the amount of strategic slowing. Notably, slowing slopes were close to flat (slope = 0 ms/trial) when the SST was conducted using a 66.67% staircase, regardless of testing environment (see Fig. 2).

It is worth noting that researchers have attempted to minimize strategic slowing by using the anticipated response version of the stop-signal task (Zandbelt & Vink, 2010). In this task, participants are required to make a response when a moving indicator on the display stops at a set target. Participants stop their response if the moving indicator stops some time before it reaches the target. By setting the response target on go trials at a fixed time point into the trial, they are unable to strategically slow their responses to avoid inhibition errors. However, there are some questions regarding the validity of SSRT measures from this task. First, many authors have demonstrated violations of context independence in the anticipated response version of the stop-signal task, which assumes that the go process will end at the same time regardless of whether or not the stop signal is presented (He et al., 2022). Second, SSRTs are both faster and less variable in this task when compared to the traditional variant (Leunissen, Zandbelt, Potocanac, Swinnen, & Coxon, 2017), which suggests that stopping in this version of the task is easier. Third, it is unclear whether stop and go processes compete in the same manner as in the traditional variant; that is, there may be less prepotent tendency towards the response given the response is already prepared (Chowdhury et al., 2020). Taking together these outstanding questions with the anticipated variant of the task, increasing the staircase stopping accuracy within the choice variant of the SST provides an alternative option for researchers attempting to minimize strategic slowing particularly for online testing environments where the experimenter may not be able to emphasize the importance of not slowing down after each block.

We found that participants completing the SST in an online testing environment produced slower SSRT estimates compared to participants in a laboratory testing environment (individual and group). However, this slowing was not specific to SSRT, as online testing also resulted in significantly slower Go RT compared to laboratory testing. Thus, these results suggest that administering a SST online with no experimenter supervision will result in overall slowing on the task that may lead to an overestimation of SSRT. Moreover, laboratory testing environments can involve the experimenter explicitly reminding participants between blocks to maintain a fast response speed. Although this was not implemented in the current study to keep instructions constant between testing conditions, this practice of prompting reminders would likely increase motivation to adhere to the task, thus reducing the incidence of strategic slowing in laboratory environments even further while motivating faster Go RT and SSRT. However, additional reminders may change the nature of control since it is externally implemented by the experiment, rather than internally motivated by the participant. Therefore, care should be taken when comparing SSRT estimates between SST administered in different testing environments. Finally, it should be noted that differences in SSRT estimation between laboratory versus online testing may be due to technical differences (e.g., hardware and software) between groups rather than motivation differences. For example, it has been demonstrated that differences in operating systems can result in different timing estimations (Bridges et al., 2020). However, the possible RT lags reported due to operating system differences are smaller than the differences in RT estimations we find between our testing environment conditions. Therefore, technical differences between groups may account for some but not all the differences we found between testing environments.

An additional advantage of the 66.67% staircase method is that, by maintaining higher stopping accuracy, it makes the SST more suitable for situations that require a greater number of stop trials, such as research focused on examining the neural mechanism underlying successful stopping (e.g., Chowdhury et al., 2019; Tran et al., 2020). For a given total number of trials, the 66.67% staircase will achieve a greater number of successful stop trials compared to that produced with the 50% staircase. Hence, using a higher percentage staircase stopping accuracy can benefit situations that require more successful stop trials but when shorter testing/assessment sessions are ideal. Examples of such situations include administering a battery of neuropsychological tests in a clinical population or conducting neuroscience research that requires a minimum sample of successful stop trials, such as in studies using transcranial magnetic stimulation (TMS), electroencephalography (EEG), functional near infrared spectroscopy (fNIRS), or functional magnetic resonance imaging (fMRI). Adopting a higher stopping accuracy can potentially have a twofold benefit of 1) saving valuable time and resources when conducting time-intensive neuroimaging experiments; and 2) improving the quality of data by managing task motivation and reducing fatigue effects associated with long testing sessions.

In summary, we found that using a 66.67% staircase produces slightly slower and comparably reliable SSRT estimates compared to using the standard 50% staircase. Further, we found that the 66.67% staircase can be a better choice when factoring in the reduction in strategic slowing and data exclusion. Past research has suggested that using a 50% staircase is suitable for unbiased estimates of SSRT (Band, 1997). Another situation where the 50% staircase would be preferred is in experiments examining failed stops and require equal sampling of successful and unsuccessful stop trials. We found that both staircases are highly correlated, and propose that a 66.67% staircase provides a suitable alternative to the recommended 50% staircase. Based on the data presented, we recommend considering a 66.67% staircase procedure over the standard 50% staircase in the following situations: 1) When experiments need to minimize strategic slowing; 2) When researchers need to collect SSRT data online with no experimenter supervision; 3) When researchers need to maintain a low exclusion rate, such as when research funds or resources are limited; and 4) When experiments require a greater number of successful stop trials. The results presented here provide researchers with information for selecting the best staircase given their constraints.

### Supplementary Information

ESM 1 (DOCX 27.4 kb)

## Appendix 1. Written instructions to participants

Please read the instructions below carefully, it will help you with the experiment.

Your main task is to respond to black arrows that appear on the screen.

Press the LEFT ARROW KEY with the right index finger when you see a LEFT ARROW and press the RIGHT ARROW KEY with the right middle finger when you see a RIGHT ARROW.

Thus, left arrow = left key and right arrow = right key.

However, on some trials (stop-signal trials) a blue square will appear after a variable delay. This indicates that you have to withhold your response.

On approximately half of the stop-signal trials, the blue square will appear soon and you will notice that it will be easy to stop your response.

On the other half of the trials, the blue square will appear late and it will become very difficult or even impossible to stop your response.

It is really important that you do not wait for the blue square to appear and that you respond as quickly and as accurately as possible to the arrows.

After all, if you start waiting for the blue square, the program will delay the presentation of the square. This will result in long reaction times.

## Footnotes

1 The mean SSRT difference between the 50 vs. 66.67% staircase was still significant when accounting for the 4.17-ms overestimation from the 66.67% staircase. This was analyzed by testing the mean SSRT difference aggregated from Experiments 1A and 1B against a value of 4.17 (t(274) = 2.22, *p* = .027, d = 0.13, BF_10_ = 0.76, error % = 0.01).

2 The within experiment test–retest reliabilities for the 50% and 66.67% staircases were .55 and .54, respectively. These correlations are lower than the correlation between the staircases but are calculated on half as much data. If one applies the Spearman–Brown correction (treating each run of the staircase as a split-half), then the reliability estimates yield .71 and .70, respectively, similar the correlation between the 50% and 66.67% staircase (.75).

## References

[CR1] Band, G. P. H. (1997). Preparation, adjustment, and inhibition of responses. Universiteit van Amsterdam.

[CR2] Bridges D, Pitiot A, MacAskill MR, Peirce JW (2020). The timing mega-study: Comparing a range of experiment generators, both lab-based and online. PeerJ.

[CR3] Chowdhury NS, Livesey EJ, Blaszczynski A, Harris JA (2017). Pathological Gambling and Motor Impulsivity: A Systematic Review with Meta-Analysis. Journal of Gambling Studies.

[CR4] Chowdhury NS, Livesey EJ, Harris JA (2019). Individual differences in intracortical inhibition during behavioural inhibition. Neuropsychologia.

[CR5] Chowdhury NS, Livesey EJ, Harris JA (2020). Stop Signal Task Training Strengthens GABA-mediated Neurotransmission within the Primary Motor Cortex. Journal of Cognitive Neuroscience.

[CR6] Coxon JP, Stinear CM, Byblow WD (2006). Intracortical inhibition during volitional inhibition of prepared action. Journal of Neurophysiology.

[CR7] He JL, Hirst RJ, Puri R, Coxon J, Byblow W, Hinder M, Puts NA (2022). OSARI, an open-source anticipated response inhibition task. Behavior Research Methods.

[CR8] Lappin JS, Eriksen CW (1966). Use of a delayed signal to stop a visual reaction-time response. Journal of Experimental Psychology.

[CR9] Leunissen I, Zandbelt BB, Potocanac Z, Swinnen SP, Coxon JP (2017). Reliable estimation of inhibitory efficiency: to anticipate, choose or simply react?. European Journal of Neuroscience.

[CR10] Livesey EJ, Livesey DJ (2016). Validation of a Bayesian adaptive estimation technique to the stop-signal task. PLoS ONE.

[CR11] Logan GD, Cowan WB (1984). On the ability to inhibit thought and action: A theory of an act of control. Psychological Review.

[CR12] Menzies L, Achard S, Chamberlain SR, Fineberg N, Chen CH, Del Campo N (2007). Neurocognitive endophenotypes of obsessive–compulsive disorder. Brain.

[CR13] Nederkoorn C, Jansen E, Mulkens S, Jansen A (2007). Impulsivity predicts treatment outcome in obese children. Behaviour research and therapy.

[CR14] Nichols SL, Waschbusch DA (2004). A review of the validity of laboratory cognitive tasks used to assess symptoms of ADHD. Child Psychiatry and Human Development.

[CR15] Oosterlaan J, Logan GD, Sergeant JA (1998). Response inhibition in AD/HD, CD, comorbid AD/HD+ CD, anxious, and control children: A meta-analysis of studies with the stop task. Journal of Child Psychology and Psychiatry.

[CR16] Tran DM, Chowdhury NS, McNair NA, Harris JA, Livesey EJ (2020). Linking cortical and behavioural inhibition: Testing the parameter specificity of a transcranial magnetic stimulation protocol. Brain Stimulation.

[CR17] Verbruggen F, Aron AR, Band GP, Beste C, Bissett PG, Brockett AT, Boehler CN (2019). A consensus guide to capturing the ability to inhibit actions and impulsive behaviors in the stop-signal task. elife.

[CR18] Verbruggen F, Chambers CD, Logan GD (2013). Fictitious inhibitory differences: how skewness and slowing distort the estimation of stopping latencies. Psychological Science.

[CR19] Verbruggen F, Logan GD (2009). Models of response inhibition in the stop-signal and stop-change paradigms. Neuroscience and Biobehavioral Reviews.

[CR20] Verbruggen F, Logan GD, Stevens MA (2008). STOP-IT: Windows executable software for the stop-signal paradigm. Behavior Research Methods.

[CR21] Vince MA (1948). The intermittency of control movements and the psychological refractory period1. British Journal of Psychology. General Section.

[CR22] Weafer J, Baggott MJ, de Wit H (2013). Test–retest reliability of behavioral measures of impulsive choice, impulsive action, and inattention. Experimental and Clinical Psychopharmacology.

[CR23] Weise L, Boecker M, Gauggel S, Falkenburger B, Drueke B (2018). A reaction-time adjusted PSI method for estimating performance in the stop-signal task. PloS one.

[CR24] Zandbelt BB, Vink M (2010). On the role of the striatum in response inhibition. PloS one.

