# Supplementary material for: The effect of staircase stopping accuracy and testing environment on stop-signal reaction time
Source: Behav Res Methods. 2023 Jan 26;56(1):500–9. doi: 10.3758/s13428-022-02058-1 (PMC9879560; doi:10.3758/s13428-022-02058-1)
Supplement: Supplementary file 1 — (DOCX 27.4 kb) [file 13428_2022_2058_MOESM1_ESM.docx]

**Supplementary Materials**

**Supplementary Table 1.** Descriptive statistics of stop signal reaction times (ms) for Experiment 1A.

|  | **50% stopping accuracy** | | | **66.67% stopping accuracy** | | |
| --- | --- | --- | --- | --- | --- | --- |
|  | **Individual** | **Group** | **Online** | **Individual** | **Group** | **Online** |
| Valid | 40 | 25 | 33 | 39 | 28 | 35 |
| Missing | 2 | 6 | 7 | 3 | 3 | 5 |
| *Mean* | 204.576 | 218.793 | 266.411 | 212.816 | 227.433 | 273.918 |
| Std. Dev. | 29.523 | 35.566 | 51.556 | 30.791 | 29.543 | 43.613 |
| Minimum | 137.75 | 165.417 | 176.417 | 156.125 | 176.063 | 181.375 |
| Maximum | 251.25 | 299.167 | 439.865 | 272.125 | 298.5 | 373.479 |

**Supplementary Table 2.** Descriptive statistics of within experiment test-retest reliability by testing environment before strategic slowing exclusion for Experiment 1B.

|  | **50% stopping accuracy** | | | **66.67% stopping accuracy** | | |
| --- | --- | --- | --- | --- | --- | --- |
|  | **Individual** | **Group** | **Online** | **Individual** | **Group** | **Online** |
| n | 57 | 66 | 71 | 55 | 68 | 64 |
| r | 0.22 | 0.52 | 0.48 | 0.43 | 0.31 | 0.46 |
| p | 0.105 | < .001 | < .001 | < .001 | 0.011 | < .001 |
